# Development of a portable hypoxia chamber for ultra-high dose rate laser-driven proton radiobiology applications

**DOI:** 10.1186/s13014-022-02024-3

**Published:** 2022-04-15

**Authors:** Pankaj Chaudhary, Deborah C. Gwynne, Boris Odlozilik, Aaron McMurray, Giuliana Milluzzo, Carla Maiorino, Domenico Doria, Hamad Ahmed, Lorenzo Romagnani, Aaron Alejo, Hersimerjit Padda, James Green, David Carroll, Nicola Booth, Paul McKenna, Satyabrata Kar, Giada Petringa, Roberto Catalano, Francesco P. Cammarata, Giuseppe A. P. Cirrone, Stephen J. McMahon, Kevin M. Prise, Marco Borghesi

**Affiliations:** 1 The Patrick G. Johnston Centre for Cancer Research, Queen’s University Belfast, Lisburn Road, Belfast, BT9 7AE Northern Ireland UK; 2 Centre for Plasma Physics, School of Mathematics and Physics, Queen’s University Belfast, Belfast, BT7 1NN Northern Ireland UK; 3 Extreme Light Infrastructure (ELI-NP) and Horia Hulubei National Institute for R & D in Physics and Nuclear Engineering (IFIN-HH), Str. Reactorului No. 30, 077125 Bucharest, Magurele, Romania; 4 Laboratoire LULI, École Polytechnique, Route de Saclay, 91128 Palaiseau, Paris, France; 5 Experimental Science Group, Central Laser Facility, Rutherford Appleton Laboratory, Didcot, Oxford, OX11 0QX England, UK; 6 Department of Physics, SUPA, University of Strathclyde, Glasgow, G1 1XQ Scotland, UK; 7 Laboratori Nazionali del Sud - Istituto Nazionale di Fisica Nucleare, Via S Sofia 62, 95123 Catania, Italy; 8 ELI-Beamlines Centre, Institute of Physics, Czech Academy of Sciences, Za Radnicí 835, 252 41 Dolní Břežany, Czech Republic

**Keywords:** Ultra-high dose rate, Laser-driven protons, Hypoxia, DNA repair

## Abstract

**Background:**

There is currently significant interest in assessing the role of oxygen in the radiobiological effects at ultra-high dose rates. Oxygen modulation is postulated to play a role in the enhanced sparing effect observed in FLASH radiotherapy, where particles are delivered at 40–1000 Gy/s. Furthermore, the development of laser-driven accelerators now enables radiobiology experiments in extreme regimes where dose rates can exceed 10^9^ Gy/s, and predicted oxygen depletion effects on cellular response can be tested. Access to appropriate experimental enviroments, allowing measurements under controlled oxygenation conditions, is a key requirement for these studies. We report on the development and application of a bespoke portable hypoxia chamber specifically designed for experiments employing laser-driven sources, but also suitable for comparator studies under FLASH and conventional irradiation conditions.

**Materials and methods:**

We used oxygen concentration measurements to test the induction of hypoxia and the maintenance capacity of the chambers. Cellular hypoxia induction was verified using hypoxia inducible factor-1α immunostaining. Calibrated radiochromic films and GEANT-4 simulations verified the dosimetry variations inside and outside the chambers. We irradiated hypoxic human skin fibroblasts (AG01522B) cells with laser-driven protons, conventional protons and reference 225 kVp X-rays to quantify DNA DSB damage and repair under hypoxia. We further measured the oxygen enhancement ratio for cell survival after X-ray exposure in normal fibroblast and radioresistant patient- derived GBM stem cells.

**Results:**

Oxygen measurements showed that our chambers maintained a radiobiological hypoxic environment for at least 45 min and pathological hypoxia for up to 24 h after disconnecting the chambers from the gas supply. We observed a significant reduction in the 53BP1 foci induced by laser-driven protons, conventional protons and X-rays in the hypoxic cells compared to normoxic cells at 30 min post-irradiation. Under hypoxic irradiations, the Laser-driven protons induced significant residual DNA DSB damage in hypoxic AG01522B cells compared to the conventional dose rate protons suggesting an important impact of these extremely high dose-rate exposures. We obtained an oxygen enhancement ratio (OER) of 2.1 ± 0.1 and 2.5 ± 0.1 respectively for the AG01522B and patient-derived GBM stem cells for X-ray irradiation using our hypoxia chambers.

**Conclusion:**

We demonstrated the design and application of portable hypoxia chambers for studying cellular radiobiological endpoints after exposure to laser-driven protons at ultra-high dose, conventional protons and X-rays. Suitable levels of reduced oxygen concentration could be maintained in the absence of external gassing to quantify hypoxic effects. The data obtained provided indication of an enhanced residual DNA DSB damage under hypoxic conditions at ultra-high dose rate compared to the conventional protons or X-rays.

**Supplementary Information:**

The online version contains supplementary material available at 10.1186/s13014-022-02024-3.

## Background

It has been known for many years that oxygen is a key radiation sensitiser and in the absence of oxygen significant radioresistance occurs which limits the effectiveness of radiotherapy [1]. Importantly, with increasing linear energy transfer (LET) the radiosensitisation by oxygen reduces even for light ions such as protons [2, 3]. Ion beam therapy using high linear energy transfer (LET) particles is recognised as an effective approach for killing radioresistant and hypoxic tumour cells [4–6]. Charged particles also provide normal tissue sparing which has enabled dose escalation for better tumour control [7–9]. Recent studies, with low LET electrons, have shown that high dose-rate approaches such as FLASH radiotherapy (typically > 100 Gy/s) are promising due to the therapeutic index boost they provide through an enhanced normal tissue sparing [10]. While the majority of the FLASH studies have used electrons, protons have also been demonstrated to be effective in sparing the normal tissues at FLASH dose rates [11] and can potentially treat deep seated tumours which is not currently feasible with FLASH electrons. FLASH results have led to a renewed interest in the radiobiological effects at high dose rates, and emerging particle sources provide today opportunities for irradiating samples at dose rates much higher than currently used in FLASH. Ultra-high dose rates (UHDR) in the range of 10^9^–10^10^ Gy/s have already been achieved by using laser-driven proton accelerators [12–15]. While most of the cellular effects at UHDR are still unknown, some effects on the DNA damage and repair have been reported including our own studies [16, 17]. A role for Oxygen depletion at high dose rates was first suggested about 50 years ago [18] and recently a number of studies have also suggested that oxygen concentration during irradiation may affect the radiobiological outcome and often result in normal tissue sparing [18–22]. A recent modelling study by Petersson et al. [23] using FLASH electrons suggested that cellular protective effects of FLASH irradiation may not be observed at atmospheric oxygen tension level as doses as high as 10–100 s Gy would be needed to deplete the significant levels of oxygen.

While studies have been performed with FLASH electrons under various oxygenation conditions [22, 24], information on the radiobiological effects of protons under hypoxia is still limited [25–28] and there are no reports at FLASH and ultra-high dose rates. This could mainly be attributed to the lack of suitable experimental systems, including appropriate hypoxia chambers, enabling such experiments with a variety of radiation sources where constraints related to physical ion beam parameters and cellular physiology make measurements difficult.

Several groups have developed hypoxia chambers for radiobiology studies but most of these chambers can be used successfully only in a horizontal orientation when placed on flat surfaces [26, 29, 30]. However, most of the experimental fixed beamlines in the cyclotron research facilities, as well as the beams produced by high power lasers within laser interaction chambers, have a horizontal orientation that allows cells irradiation only in a vertical position i.e. perpendicular to the beam. In this situation, horizontal chambers may not be suitable as there is a high chance of liquid medium spillage and mixing when multi-well plates or petri-dishes are used. The permanent mounting of hypoxia incubators on a beam line is impractical in many situations due to dosimetry requirements, and a simple lightweight, gas-impermeable, portable chamber capable of maintaining hypoxic environment for long durations would be beneficial. Most of the hypoxia chambers used previously either relied on continuous hypoxia gas supply during irradiation or gassing before irradiation. Re-oxygenation of the chambers upon disconnecting from the gas supply or over time can significantly impact the radiobiological response, as radiobiological hypoxia oxygen concentration is less than equal to 0.4% oxygen and values above this could be reached rapidly. Metsällä et al. [31] have developed a portable hypoxia chamber equipped with a gas cylinder for controlling multiple samples at a time. Walter et al. also demonstrated the use of a hypoxia chamber for studies with high energy carbon, oxygen and nitrogen ions [26, 32]. While their hypoxia chamber system was effective for high energy particles, the chamber was not tested for the lower energies of current relevance to laser-driven protons, where the particle energies used for cellular radiobiology endpoints are often typically ~ 10–20 MeV, with short ranges.

The procedures and constraints of laser driven proton irradiation are currently very different than conventional proton beam irradiations using RF accelerator beams. The workflow in a laser-driven proton irradiation procedure is not as fast as in a conventional proton beam experiment. The proton beam is generated inside a high vacuum laser interaction chamber by focussing very high power lasers onto thin targets. Biological samples cannot be irradiated inside the interaction chamber due to the high vacuum (< 1 × 10^−4^ mbar) so, in a typical set-up [16], the beam needs to be steered out of the interaction chamber using strong magnets, and exits the chamber through a thin plastic window (typically Kapton). This Kapton window acts as a sample irradiation port and is kept isolated from any outside elements by installation of a narrow diameter (20 cm) sample re-entry tube perpendicular to the axis of the exit window. This limits the size of cell samples which can be irradiated and only compact hypoxia chambers can be used to control the gassing conditions. The compact hypoxia chambers are introduced on a rail through this narrow tube such that the hypoxia chamber window can align with the Kapton window during the irradiation. Target alignment and radiation safety checks required before each shot significantly increase the set-up time required to prepare for and complete an irradiation compared to conventional proton exposures. The cells, attached as monolayers in the Mylar dishes, cannot be held in upright position for long durations as this may lead to their dehydration. To overcome this problem, the hypoxia chambers can be mounted on remotely controlled motorized flippers. Immediately before irradiation, the hypoxia chambers are slowly flipped vertically and after irradiation they are returned to the horizontal position, removed and taken for post irradiation processing. We addressed all these issues during the design and development of a portable hypoxia chamber and successfully used it to measure ultra-high dose-rate laser-driven proton DNA damage and repair under oxic and hypoxic conditions alongside comparator studies with conventional dose rate protons and X-rays. Furthermore, we also measured the oxygen enhancement ratio (OER) for survival of radioresistant patient derived Glioblastoma (E2) and normal human skin fibroblast AG01522 cells irradiated with reference X-rays in the hypoxic chambers to validate the radiobiological impact of irradiating two radiobiologically variable cell lines under hypoxia. Overall, our results clearly indicate the effectiveness of these hypoxia chambers in the maintenance of a hypoxic environment during irradiation with various radiation qualities, without adversely affecting the physical and radiobiological readouts.

## Materials and methods

### Cells and culture

Both human cell lines used in this study were authenticated through STR profiling at European Collection of Authenticated Cell Cultures (ECACC) operated by Public Health England, United Kingdom. AG01522B cells were obtained from the Coriell Institute for Medical Research (Camden, New Jersey, USA) and maintained in α**-**modified Minimum Essential Medium (MEM) (Sigma Aldrich) supplemented with 20% Fetal Bovine Serum (FBS) and 1% penicillin–streptomycin (Thermo Fischer Scientific, Loughborough, UK). These cells were routinely tested for Mycoplasma contamination and have a finite life span. For all our experiments, early passage cells within passage 2–4 after procuring from the Coriell Repository. Glioblastoma stem like cells (E2 cells) were kindly provided by Prof. Anthony Chalmers (University of Glasgow, UK) and have been previously characterized for the expression of stem cell biomarkers such as NG2, Olig2 and Sox-2. These cells are cultured in Advanced Dulbecco’s Modified Eagle’s Medium/F-12 serum free medium, supplemented with B27, N2, L-Glutamine, heparin, epidermal growth factor and basal fibroblast growth factor (Thermo Fischer Scientific, Loughborough, UK) and were also routinely tested for mycoplasma contamination. Cell culture flasks and cell dishes used for E2 cells were coated with a thin layer of growth factor reduced basement membrane Matrigel (Corning, NY, USA) dissolved in DMEM/F-12 medium at a dilution of 1:40 giving a final concentration of matrigel proteins of 0.225 mg/ml of medium. All the cells were maintained in 5% CO_2_ with 95% humidity at 37** °C**.

### Hypoxia chamber design, hypoxia induction and Oxygen measurement

**Fig. 1:**
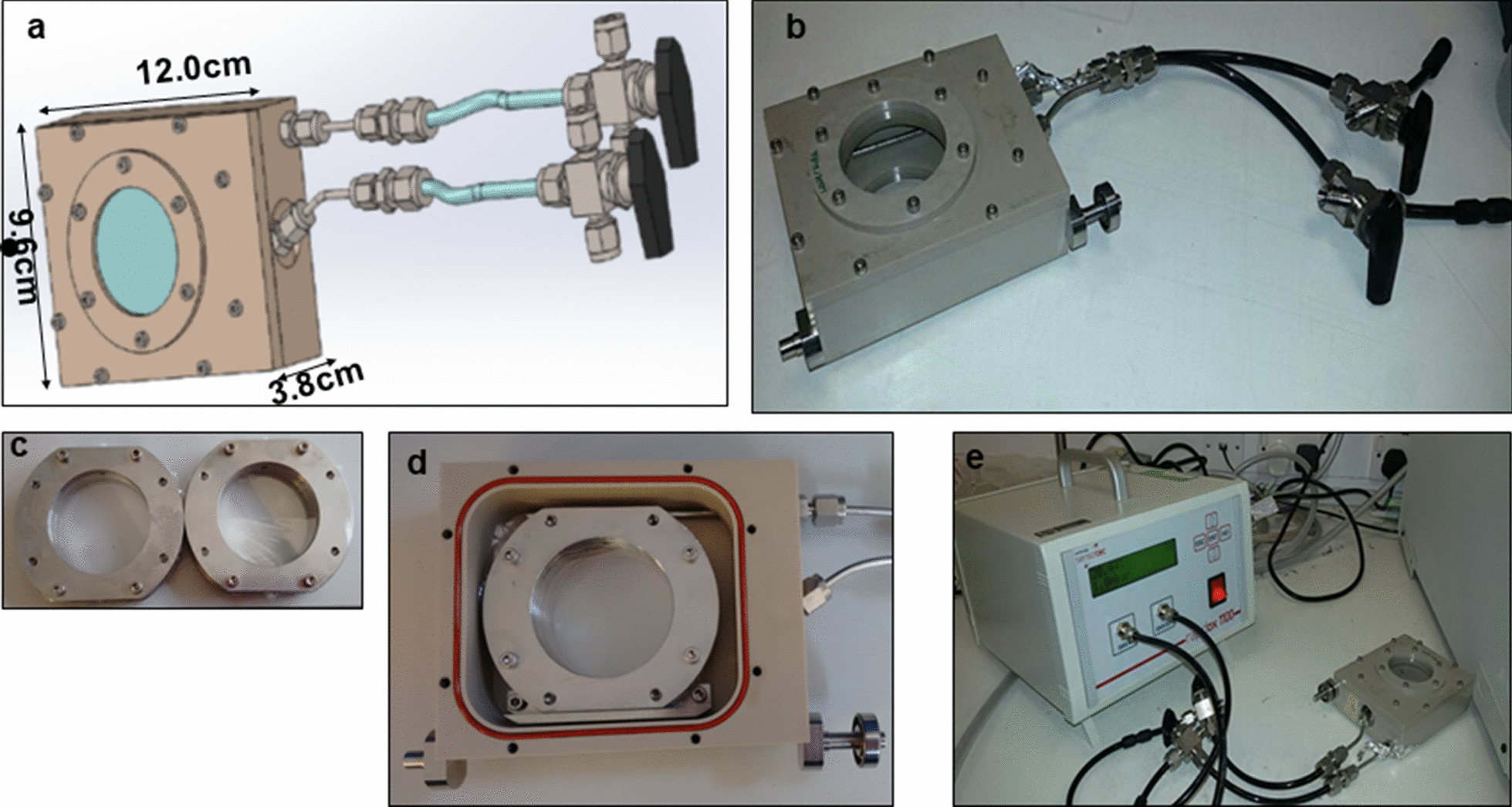
**a** Schematic of the hypoxia chamber. **b** Manufactured hypoxia chamber with the inlet and outlet valves connected to the tubing. Gas-impermeable 12 µm transparent PVDC window allows visual alignment and irradiation with low energy protons. **c** Stainless-steel dishes mounted with 3 µm Mylar for growing cells in monolayers and irradiating them with low energy protons. **d** Assembly of the stainless-steel dish inside the hypoxia chamber which can be sealed with a lid and mounted on a motorized stage. **e** Arrangement used for measuring the oxygen concentrations over time after gassing the hypoxia chambers using a Rapidox 1100Z detector (Cambridge Sensotec, Cambridge UK)

The design and assembly of the hypoxia chamber is shown in Fig. 1a–e. The main body of the hypoxia chamber is a hollow box made of polyetheretherketone (PEEK) (Direct Plastics, Sheffield, England, UK) sheet of 5 mm with dimensions of 12.0 cm (length) 9.6 cm (width) and 3.8 cm (height). The front and rear faces of the chamber have 4.7 cm diameter circular openings. These openings are sealed with a 12.5 µm oxygen impermeable polyvinylidene chloride (PVDC) or Saran membrane (Goodfellow Cambridge Ltd, Huntingdon, England), attached with 0.4 cm and 0.2 cm thick PEEK rings screwed with silicone ‘O’ rings underneath for sealing on both the front and rear, creating two transparent windows. Two stainless steel gas ports, one inlet and one outlet, were attached to the right side of the box. These ports were connected to two-way valves with flexible polyurethane tubing. The inlet port extends inside the chamber to the left-hand side of the box to ensure uniform thorough saturation of the chamber with a hypoxia gas mixture.

Oxygen concentration measurements were taken for six independent chambers using an oxygen gas analyser (Rapidox 1100Z, Cambridge Sensotec, Cambridge UK) connected to the chambers in the arrangement shown in Fig. 1e. Briefly, the chambers were flushed with the hypoxic gas (95% N_2_, 5% CO_2_, BOC Gases, Belfast, UK), for 15 min through the inlet port. The outlet from the hypoxia chamber was fed into the inlet port of the oxygen analyser and the outlet of the analyser was connected to a small water-filled flask for visual monitoring of the gas flow rate. The chambers were gassed at a flow rate of 0.5–1 L per minute until the oxygen saturation dropped down to 0.005–0.01% between 0 and 3 mm Hg oxygen tension. At this stage, the inlet and outlet valves of the chambers were closed, the oxygen analyser outlet tube was immediately removed from the water-filled flask and attached to the inlet port of the chamber. In this way a closed loop was formed between the hypoxia chamber and the oxygen gas analyser that allowed continuous monitoring of the oxygen concentration inside the chamber for the next 24 h.

For hypoxia induction, the cells were grown inside customized stainless steel dishes with a 3 µm Mylar membrane base for cell attachment (Fig. 1c) which was mounted inside the sterile (thoroughly wiped with 70% ethanol and dried in the laminar air flow) chamber (Fig. 1d) in less than 5 min under the sterile air of the laminar flow hood. The whole assembly was then placed in a cell culture incubator at 37 °C where the chambers were continuously gassed for 4 h with the hypoxia gas mixture. To quantify the initial change in oxygen concentration, oxygen concentration data from all chambers were pooled together and fitted using an equation of the form $C = C_{0} \left( {1 - e^{ - kt} } \right)$, where $C_{0}$ is a plateau concentration and $k$ is a rate constant describing the rate of reoxygenation. The flexible fittings used in the hypoxia chambers make them suitable to connect more than six hypoxia chambers in series easily, the only limitation being the size of the incubator holding the chambers at the optimum temperature for gassing. For larger experiments, one can, in principle, scale up the set up without any technical issues, although combinations using more than 12 hypoxia chambers may need further verification e.g. to test whether the pressure inside the hypoxia chambers at the beginning or the end of the series is similar or significantly variable.

### Irradiations

#### Laser-driven protons

**Fig. 2:**
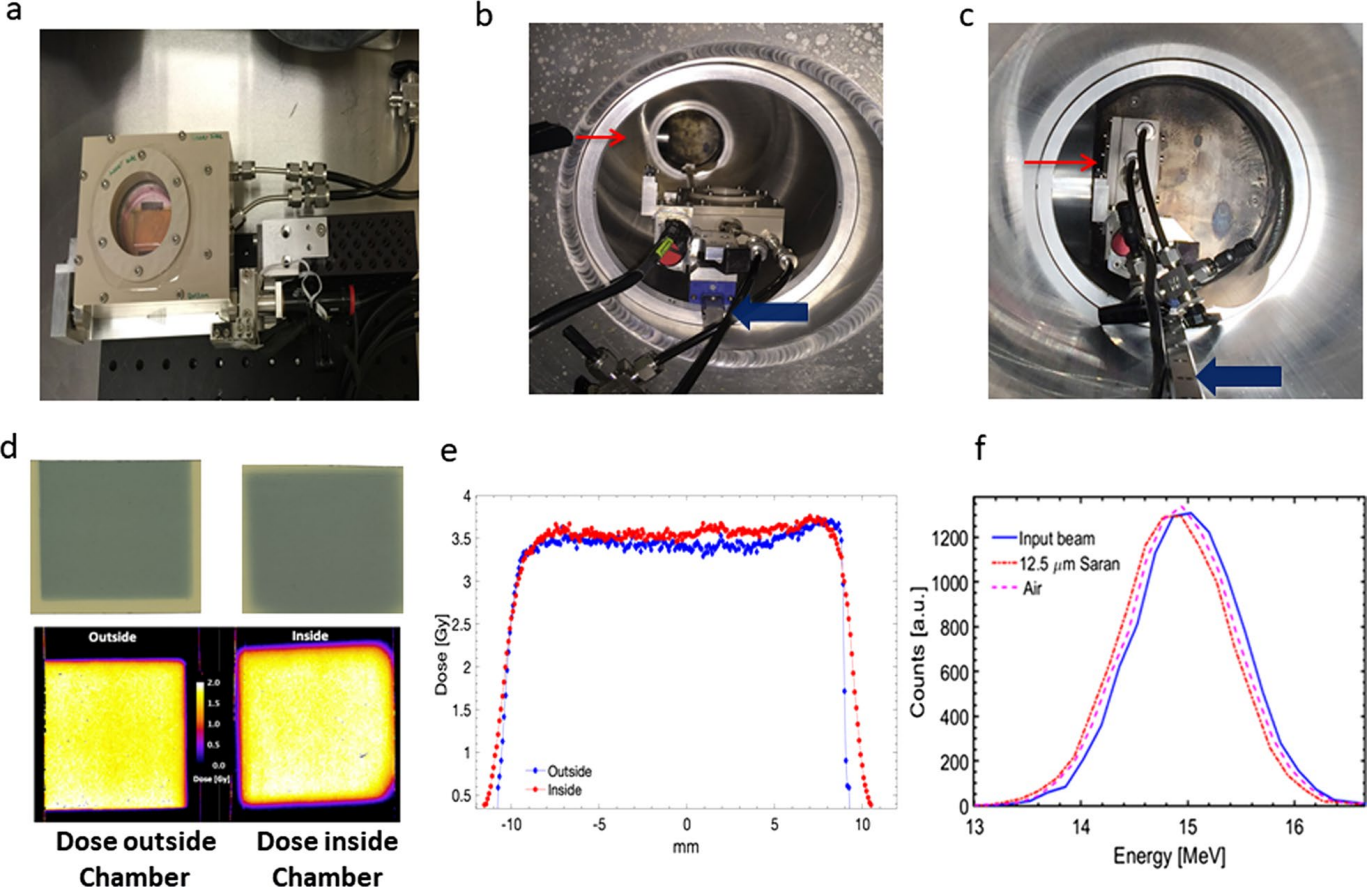
**a** Hypoxia chamber assembled with the cell dish. **b** Irradiation set up of hypoxia chamber inside the re-entry tube. The chamber is slid on the metal rail (blue arrows) towards the end of the tube and just before and during radiation the chamber is tilted vertically as shown in **c** using a motorized mount such that the transparent PVDC window of the hypoxia chamber is facing the Kapton window through which the laser-accelerated proton beam emerges and irradiates the cells grown on the Mylar mounted in a stainless steel dish inside the hypoxia chambers. **d** Comparison of EBT3 films irradiated with a 30 MeV proton beam outside and inside the chamber **e** Lineout across dose profiles in (**d**). **f** Energy spectra of 15 MeV proton beam (blue curve) crossing the 2 cm of air gap inside the chamber with (red line) and without (dashed pink line) the 12.5 μm Saran window of the hypoxia chamber, obtained using the GEANT 4 Monte Carlo Simulation

Laser-driven protons were generated at the Petawatt arm of the VULCAN laser at the Central Laser Facility of the Rutherford Appleton Laboratory, Didcot, Oxford, UK. Protons were accelerated by focussing the Vulcan Laser at an intensity of order 5 10^20^ W/cm^2^ onto a 25 µm-thick aluminium foil. The protons were accelerated through the Target Normal Sheath Acceleration mechanism [33, 34] from the hydrogen contained in a contaminant layer on the rear surface of the target. As typical for this mechanism, the energy spectrum of the accelerated protons was broadband, approximately decreasing exponentially, up to a cut-off energy of ~ 30 MeV. The proton irradiation setup was designed to minimize the distance between the target and the cells, compatibly with energy selection and shielding requirements, in order to achieve a suitable dose on the cells on a single-shot basis, as well as to minimize the temporal duration of the ion irradiation. A 1.0 T magnet was used in conjunction with a collimator and pinhole to disperse and spatially select the proton energy and irradiate the cells with the selected 15 ± 1 MeV protons. The protons exited the interaction chamber through a flange-mounted 50 µm Kapton window before reaching the cells, located approximately 30 cm away from the laser interaction point at the transverse position corresponding to the 15 MeV proton spatial dispersion. Dose rates of 2 × 10^9^ Gy/s were achieved in single ion pulses of ~ 400 ps duration. Hypoxia chambers with motorised mounts were inserted on a slide rail through a re-entrant tube in a horizontal position to keep the cells submerged in liquid medium. Just prior to firing the laser, the hypoxia chambers were raised slowly from the horizontal to vertical position, so that the cells, without any cell culture medium layer in front, were precisely located in the beam path, through a remotely controlled motorized mount as shown in Fig. 2a–c.

### Conventional dose rate protons

Cells were irradiated at a conventional dose rate of 4 Gy per minute with the proton beams accelerated by the superconducting cyclotron (CS) along the CATANA proton therapy beam line of Laboratory Nazionale del Sud (LNS), Istituto Nazionale Fisica Nucleare (INFN) Catania, Italy. The 30 MeV proton beam extracted from the CS, was then degraded using a 4 mm-thick PMMA range shifter placed in-air along the beam line, in order to obtain 15 MeV protons at the cell position. The portable hypoxia chambers were mounted inside the Perspex sample holder and aligned on a motorized X–Y translator stage as shown in supplementary figure-1, and movement across X and Y axis was controlled remotely. A 2 cm × 2 cm collimated proton beam irradiated the cells inside the hypoxia chambers and nine collimated fields were used to fully irradiate the cell dish.

### X-rays

Cellular irradiations with 225 kVp X-rays were performed in house in our institution using a X-Rad 225 (Precision X-ray, Connecticut, USA), X-ray generator at a dose rate of 0.59 Gy/min. Both hypoxic and normoxic cells were irradiated inside the shielded cabinet for the time duration required to deliver each dose.

### Dosimetry and simulations for hypoxia chambers

Before irradiating the cells, we first performed dosimetry inside and outside the blank hypoxia chambers using EBT3 Radiochromic films, which are known to show a dose-rate independence for dose rates as high as 10^10^ Gy/s [35, 36], to test for any dose variations (shown in Fig. 2d, e). The horizontal dose profiles measured with the EBT3 film placed at the cell position inside the chamber (red data points) and outside the chamber (blue data points) are shown in Fig. 2e for comparison. During the cellular irradiations employing laser-driven protons, the dose delivered to the cells placed within the hypoxia chamber was measured, for every irradiation, using EBT3 type films placed just after the dish, i.e. behind the 3 µm Mylar foil where the cells were attached. This setup allowed measurement of the dose in a position very close to the position of the cells. The EBT3 Radiochromic films had been previously calibrated with a 35 MeV clinical proton beam conventionally accelerated at the CATANA beam line of the LNS-INFN. Using the dose calibration, and exploiting the demonstrated dose-independence and high spatial resolution of EBT3, it was then possible to reconstruct the absolute dose delivered per shot with a 10% uncertainty at any point of the irradiated region of the cell dish. A Monte Carlo GEANT4 simulation was also performed to estimate the proton energy loss through all the components traversed within the hypoxia chamber, considering as the input a narrow band 15 MeV proton beam impinging into the 12.5 µm PVDC Saran window. The energy loss is not significant as shown in Fig. 2f. We also used EBT3 films for dosimetry of X-rays, which we mainly used to irradiate the cells for cell survival and oxygen enhancement ratio studies as shown in the Additional file 1: Fig. S1. As X-rays dosimetry is well characterised and reproducible, no sample to sample measurement was undertaken.

### Biological validation of hypoxia

The hypoxia inducible factor -1α (HIF-1α) is a well-known biomarker of hypoxia which is expressed upon hypoxia induction in human cells. Using immunofluorescent staining we detected HIF-1α in human skin fibroblasts after hypoxia induction using our chambers. Briefly, the cells were incubated under the hypoxic gas mixture flow for 4 h and immediately fixed in 4% paraformaldehyde. Cells were then permeabilized, blocked in 10% goat serum and probed with a mouse primary anti-HIF-1 α antibody (Abcam, Cambridge, UK) and then washed and probed with goat-anti-mouse Alexa flour 594 secondary antibody (ThermoFischer Scientific, UK). Finally, the cells were mounted with antifade reagent containing nuclear stain DAPI.

### DNA DSB damage and repair under hypoxia

We detected DNA DSB damage and repair using the 53BP1 foci formation assay in hypoxic and oxic AG01522B cells irradiated with laser-driven protons, conventional protons and X-rays. Cells were gassed for four hours and then irradiated with 1 Gy of either15 MeV laser-driven protons, conventional dose rate protons or X-rays. The cells were then fixed in 4% paraformaldehyde (PFA) for 20 min (room temperature) at 0.5- and 24-h post-irradiation. For co-staining with HIF-1α and 53BP1, after fixation the samples were rinsed with PBS (Phosphate Buffered Saline) and later permeabilized in 0.5% Triton X-100 (Sigma Aldrich) in PBS for 10 min at room temperature and subsequently blocked in 2 ml of blocking buffer (10% goat serum and 0.25% Triton X-100 in PBS) at 37 °C for 2 h. After blocking, 1 ml of the primary antibodies mixture, 1:1000 53BP1 (Novus Biologicals, Littleton, CO, USA) and 1:500 HIF-1α (Abcam, Cambridge, UK) diluted in the blocking medium was added to the dishes and incubated at 37 °C for 1 h and then washed three times in PBS containing 0.1% Triton X-100. The cells were then probed with a mixture of secondary antibodies (goat anti-rabbit-Alexa Fluor 488 and goat anti-mouse-Alexa Fluor 594), at a dilution of 1:1000 respectively in blocking buffer and incubated for 1 h at 37 °C. The samples were then washed and mounted on coverslips using an anti-fade reagent containing DAPI. Cells were then scored for 53BP1 foci in both oxic and hypoxic samples and plotted as mean number of foci per cell for 0.5 and 24 h time points.

#### Oxygen enhancement ratio

We used the clonogenic cell survival assay to calculate the oxygen enhancement ratio for normal human skin fibroblasts (AG01522B) and patient derived radioresistant glioblastoma stem like cells (E2 Cells) irradiated with X-rays. As these two cell lines have variable intrinsic radiosensitivity, the X-ray dose response under hypoxia for these cell lines enabled further validation of the chambers’ ability to maintain radiobiological hypoxia. Due to the technical difficulties involved in generating radiation doses in the range relevant for cell survival assay, it was not feasible to conduct cell survival assay with laser-accelerated protons during these studies and for this objective, we limited our experiments to X-rays only. Both the normal and radioresistant cells were plated at a density of 2 × 10^5^ cells per dish on Mylar and incubated for 24 h. After 24 h, the cell culture medium was replaced with fresh medium and the dishes mounted inside hypoxia chambers connected to the hypoxia gas supply as described previously. After 4 h of gassing, the valves were closed, the chambers disconnected, and hypoxic cells exposed to various doses of X-rays. Immediately after irradiation, each chamber was opened, and the cells were dissociated and plated following the clonogenic assay protocol [37]. After twelve days we quantified the colonies in each well of the six-well plates, plotted dose response curves and calculated oxygen enhancement ratio at doses resulting in 10% (D_10_), 50% (D_50_) and 90%(D_90_) surviving fraction for both cell lines to determine the impact of both low and high doses of X-rays, according to the formula

$${\text{OER}} = {\text{D}}_{{{1}0,{5}0{\text{ or 9}}0}} \left( {{\text{Hypoxic}}} \right)/{\text{D}}_{{{1}0,{5}0{\text{ or 9}}0\% }} \left( {{\text{Oxic}}} \right)$$

where D_10, 50 or 90%_ = dose resulting in 10, 50 or 90% surviving fraction.

### Data analysis and statistics

Oxygen measurements were carried out using six independent chambers and data is shown for individual chambers as well as the mean for six chambers for comparison. 53BP1 foci were analysed in at least 100 cells in three replicates and reported as mean values ± standard error of the mean (SEM). Statistical significance analysis comparing the foci induction and cell survival values under oxic and hypoxic conditions was performed using an unpaired T test available in GraphPad Prism software, version 9.1.2 (LaJolla, CA,USA), with a threshold for significance at *P* < 0.05 and P < 0.01. For OER calculations, cell survival data from at least two independent X-rays dose response replicates in AG01522B and E2 cells were obtained and fitted in modified Linear Quadratic fitting of Graphpad Prism software. Various transformants reporting dose resulting in 10% cell survival (D_10_), 50% cell survival (D_50_) and 90% cell survival (D_90_) were obtained from the fits with 95% confidential intervals (CI). The obtained values for D_10_, D_50_ and D_90_ under hypoxia were divided with the D_10_, D_50_ and D_90_ values obtained under normoxic conditions to obtain OER and shown as OER with 5% Error.

## Results

### Oxygen concentration measurement

**Fig. 3:**
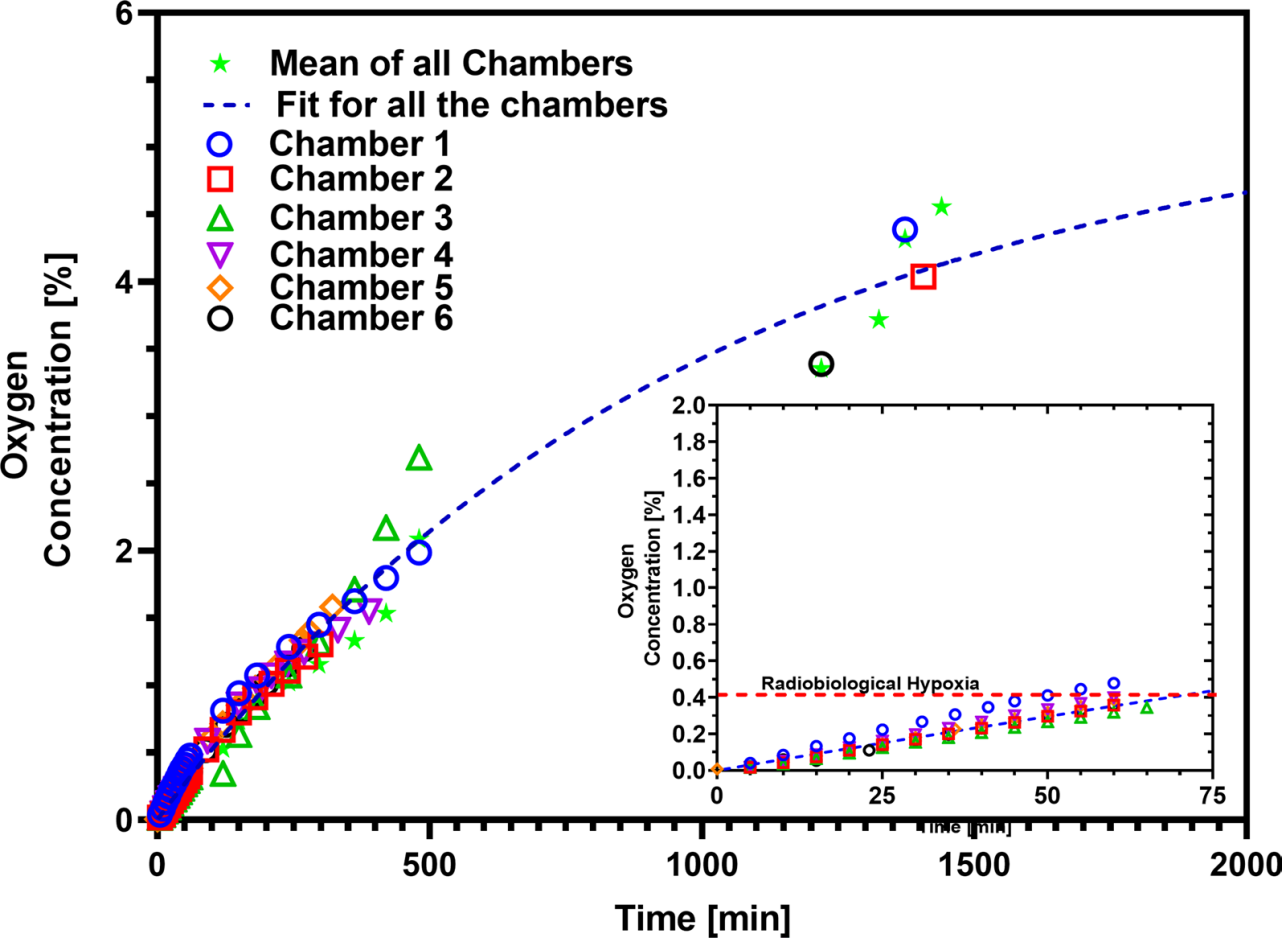
Physical validation of hypoxia as carried out using an Oxygen sensing probe for measuring the Oxygen concentration measurement over the period of 24 h after gassing the six hypoxia chambers. The inset graphs shows the oxygen concentration during the first 75 min. For the initial 45 min after gassing, all the chambers maintained the oxygen level below 0.4% (radiobiological hypoxia shown by red-dashed line). The plot aslo shows the average of all chambers (bright green stars) and one phase association reoxygenation fitting is shown in blue dashed line. At later time points (24 h of gassing) some deviation in the oxygen concentration was also observed

We measured oxygen concentrations in six individual hypoxia chambers as shown in Fig. 3. All six chambers maintained a physiological hypoxic environment (≤ 2% O_2_ [38]) for up to 4 h after disconnection from the gas supply. These chambers maintained radiobiological hypoxia (≤ 0.4% Oxygen [38]) for at least 45 min after disconnection from the gas supply, which provided sufficient time to irradiate and process the samples under radiobiological hypoxia conditions without the risk of re-oxygenation. When data for all chambers was fitted using a single curve, good agreement was found for the first six hours, where the oxygen concentrations remained below 2%. The best-fitting curve (line in Fig. 3) was in agreement with all chambers with a mean absolute deviation of 0.06% O_2_. The best-fitting parameters were $C_{0} = 4.9 \pm 0.2\%$ O_2_ and $k = 0.0012 \pm 0.0001$ min^−1^. For the early time period, this is equivalent to a reoxygenation rate of $0.00588 \pm 0.0005$% O_2_/min. Chamber 3 showed some deviations from the rest of the chambers after 4 h of gassing. One significant outlier point was excluded from this analysis, for chamber 5 at 24 h, which showed an O_2_ concentration of almost 7%, compared to 4% for the other chambers. This suggests there may be some greater variability at later time points, but that these chambers are suitable for maintaining radiobiological hypoxia over practical experimental timescales of at least 45 min which is sufficient to irradiate the cells while under radiobiological hypoxia and transport them back to the biology laboratory for further processing.

### Biological verification of hypoxia using HIF-1α

**Fig. 4:**
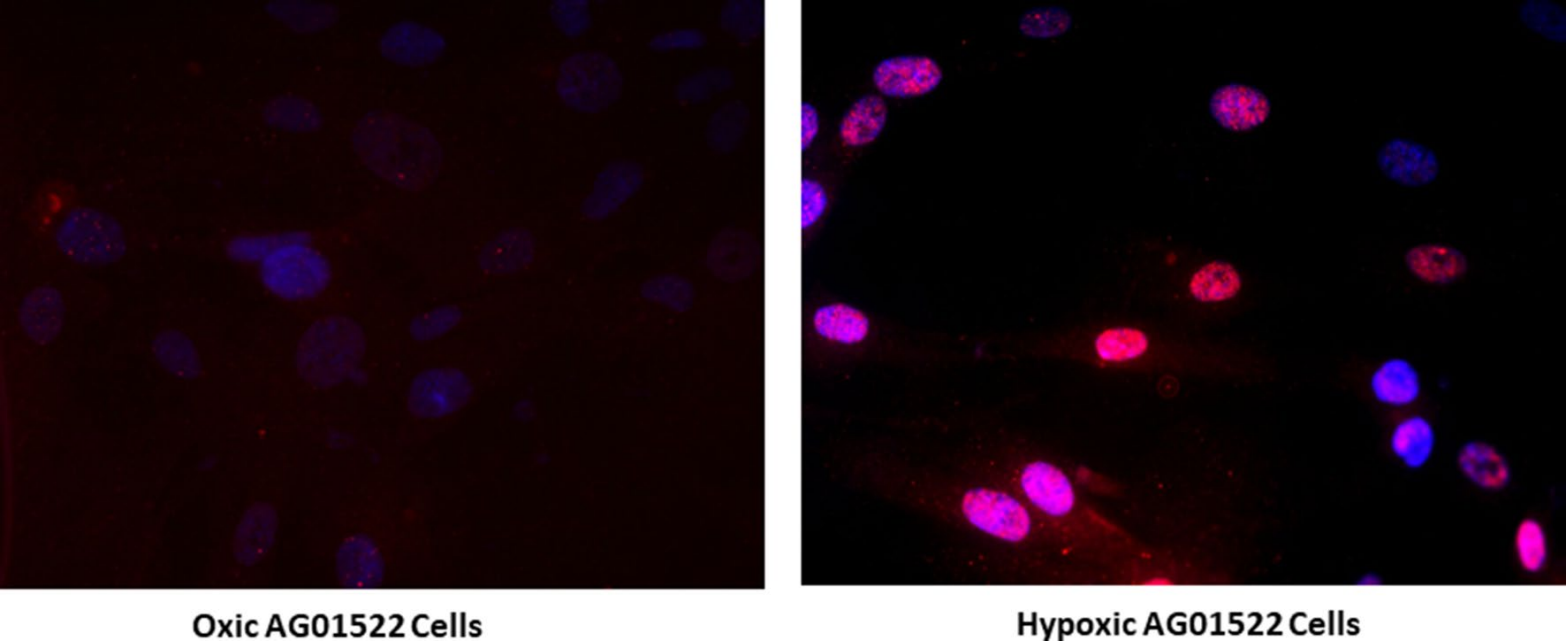
Immunofluorescent detection of hypoxia induction in AG01522B cells after 4 h of gassing with 95% nitrogen and 5% CO_2_ inside hypoxia chambers. HIF-1 α was detected using primary anti-HIF-1α antibody later probed with secondary Alexa fluor 594 antibody

We used an immunofluorescent assay to detect HIF-1α in the AG01522B cells after 4 h hypoxia induction. As shown in Fig. 4, HIF-1α is mainly localized in the nucleus of hypoxic cells while in the oxic samples only faint cytoplasmic staining is seen. We observed clear differences in the intensity of HIF-1α staining under both hypoxic and oxic conditions after four hours of gassing for hypoxia induction and similar changes were also noticed in the cells co-stained with DNA DSB marker 53BP1 and HIF-1α.

### DNA DSB damage induction and repair in hypoxic cells

**Fig. 5:**
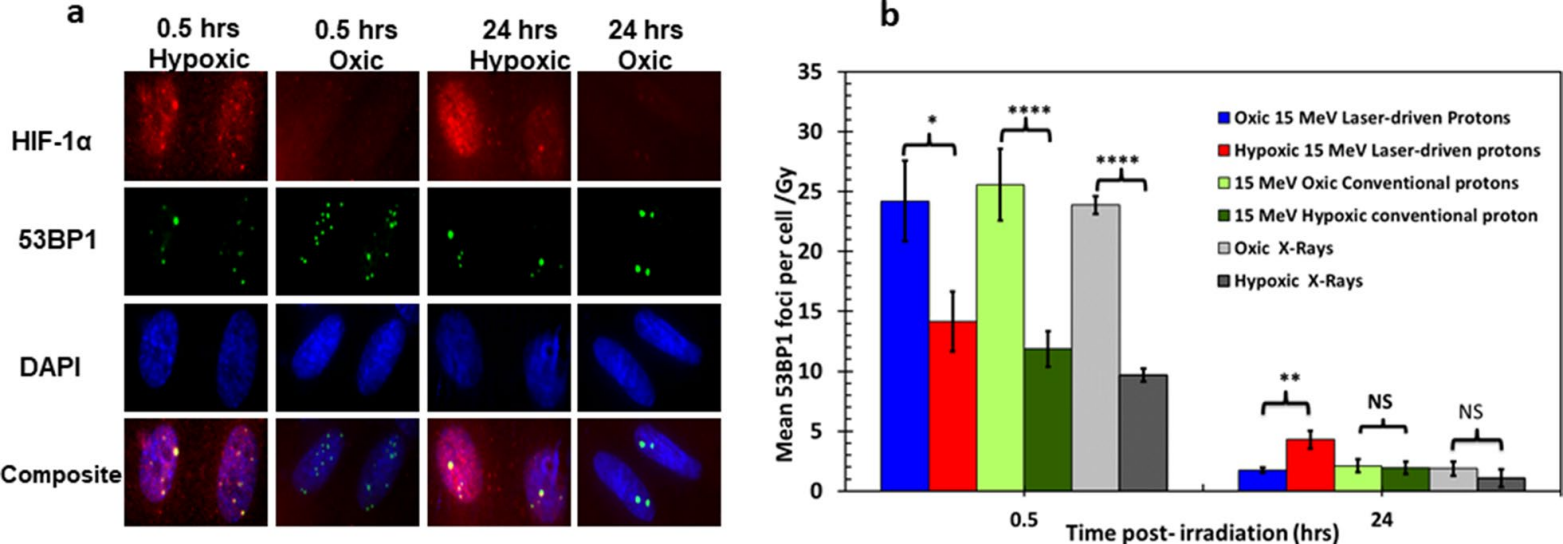
**a** Laser accelerated 15 MeV protons induced DNA DSB damage and repair detection using 53BP1 foci formation assay in AG01522B cells irradiated under hypoxic and oxic conditions. Cells were incubated under hypoxia for 4 h, irradiated, then later fixed and stained for 53BP1 foci (green) and HIF-1α (in red). **b** Quantification of laser -accelerated protons induced 53BP1 foci under oxic and hypoxic conditions for comparison cells in similar conditions were also irradiated with 1 Gy of 225 kVp X-rays. All the values on the graphs are shown after substracting the background control values. For each data point at least 100 cells in duplicate slides were analyzed and data is shown as an average of two independent replicates. Error bars represent the standard error of the mean. Statistical significance was analysed using Student’s un-paired *T* test and * represents *P* values ≤ 0.05, *** represents *P* values ≤ 0.0001; NS-non-significant

53BP1 foci formation is regarded as one of the hallmarks of DNA DSB damage along with γ-H2AX [39–42]. We detected 53BP1 foci formation in AG01522B cells irradiated with 15 MeV laser-driven protons, conventional protons and X-rays with simultaneous staining of HIF-1α as shown in Fig. 5a where the 53BP1 foci are shown in green, HIF-1α in red and nucleus in blue. Quantitative analysis, as shown in Fig. 5b clearly indicates the effect of hypoxia on the DSB damage induction. Under oxic conditions, the levels of initial damage at 0.5 h induced by laser-driven protons, conventional protons and X-rays were similar, with mean 53BP1 foci per cell values of 24 ± 3, 25.6 ± 3 and 24.9 ± 0.8 respectively. Similarly, under hypoxia, the levels of initial DNA DSB damage were similar for laser-driven protons, conventional dose rate protons and X-rays with mean 53BP1 foci levels of 14.16 ± 2.5, 11.9 ± 1.5 and 10 ± 1. Overall, a decrease in the mean 53BP1 foci per cell clearly show the impact of cellular hypoxia on the DNA DSB damage yields.

At 24 h post-irradiation, we detected significant changes (*P* < 0.05) in the mean number of residual 53BP1 foci induced by laser-driven protons under hypoxia with mean foci values of 4.5 ± 0. 8 compared to mean values of 1.9 ± 0.5 and 1.8 ± 0.2 for conventional protons and X-rays respectively. The residual foci measured for both conventional protons and X-rays were not significantly different from each other. We further compared the DSB foci induced by laser-driven protons and conventional protons by normalizing 53BP1 foci induction value at 0.5 and 24 h to those induced by X-rays defined as relative foci induction (RFI) as the ratio of foci induction by laser-driven or conventional proton to X-rays induced foci at same time point as shown in Additional file 1: Fig. S2. Laser-driven protons showed a statistically significant (*P* < 0.05) RFI value of 2.4 ± 0.2 compared to the conventional proton RFI value of 1.08 ± 0.1 for the residual (24 h) 53BP1 foci under hypoxia.

### Oxygen enhancement ratio in AG01522B and patient-derived GBM stem cells

**Fig. 6:**
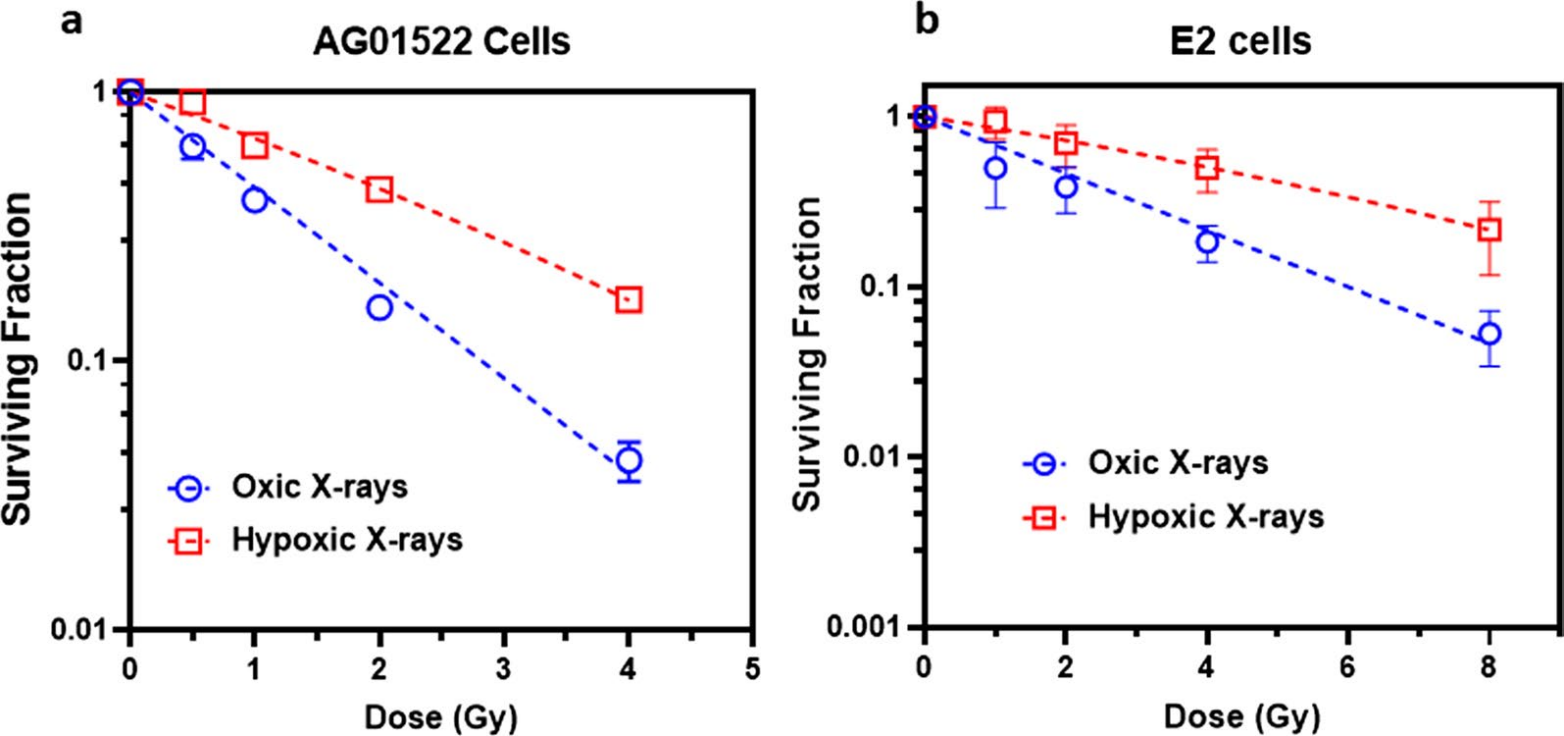
**a** X-rays Dose response curve of human normal skin fibroblasts (AG01522B cells) and **b** patient derived glioblastoma stem cells (E2 cells) obtained using clonogenic assay. For OER calculation various dose values resulting in surviving fraction of 10, 50 and 90% (D_10_, D_50_ and D_90_) were obtained as transformants on the surviving curves under oxic and hypoxic conditions. The values obtained for various doses were used to calculate OER for X-rays in both AG01522B and E2 cells as shown in the Additional file 1: Table S1

We calculated the oxygen enhancement ratio for cell survival of X-rays in both the normal AG01522B cells and E2 cells. Figure 6a shows the AG01522B cell survival curve for oxic and hypoxic conditions and Fig. 6b shows the cell survival curves for E2 cells under oxic and hypoxic conditions. Dose resulting in cell survival at various levels such as D_10_, D_50_ and D_90_ were obtained for each cell line from the X-rays dose response curve under oxic and hypoxic conditions. The OER varied for each cell line and a dose dependent variation in OER was observed as shown in Additional file 1: Table S1. For normal human fibroblast cell line AG01522B cells we observed an OER value of 1.80 ± 0.09, 2.0 ± 0.1 and 2.2 ± 0.1 respectively for D_10_, D_50_ and D_90_. For radioresistant E2 cells the OER values were 1.80 ± 0.09, 2.2 ± 0.1 and 2.5 ± 0.1. The OER values varied both with the change in dose and the nature of cell lines. Variation in OER as a function of dose and cellular radiosensitivity are expected as also reported in [42, 43].

## Discussion

Overcoming hypoxic radioresistance still remains one of the most important unmet challenges even with the most advanced radiotherapy modalities [44]. Despite the tremendous progress made in imaging hypoxia [45, 46] and radiotherapy modelling based concepts such as dose and LET painting with charged particles [5, 47, 48], the treatment of hypoxic tumours is still challenging. Radiobiological data on the effectiveness of charged particles under hypoxia is also still very limited. At UHDR above 10^7^ Gy/s, only a few papers are available that show the role of oxygen depletion with electron beams [22] while there is no radiobiological data reported with UHDR protons. In this study, we successfully designed and developed compact portable hypoxia chambers and tested them for maintaining radiobiological hypoxia for extended periods after gassing and being disconnected from the gas supply. As laser-driven proton beam acceleration techniques are still evolving, cellular irradiation with laser-driven beams is not yet optimal in terms of—dose stability and the ability to raster scan at speed across a sample as possible with cyclotron accelerated proton beams. On the other hand, the achievement of UHDR exceeding 10^8^ Gy/s in single pulses of ~ ns duration makes this an ideal approach to access and study novel radiobiological regimes.

Due to current constraints at high-power laser facilities, the time between irradiation of individual samples can vary from several minutes to an hour. Our hypoxia chambers facilitate the maintenance of hypoxia for the entire duration of transportation, alignment, irradiation and transport back to a biological laboratory for post-irradiation processing. Oxygen concentration values relevant to radiobiological, pathological and physiological levels of hypoxia and the hypoxia retention efficiency at all levels of the chambers are shown in Fig. 3. All six hypoxia chambers were able to maintain a physiological hypoxia environment (≤ 2% O_2_) for the full 24 h’ test period and radiobiological hypoxia for about 45 min after disconnecting from the gas supply.

Hypoxia systems, currently in use, offer the capability of irradiating up to 6 samples simultaneously in a six-well plate format [31]. While this is advantageous to speed up the workflow, a possible downside is that, in case of any issue with the irradiation, all of the six samples are used. Our hypoxia chamber can be used to irradiate single samples one at a time, which minimizes the risk of sample wastage especially in case of laser-driven ions where the dose variation can be significant from sample to sample [49]. In addition, for multiple-sample hypoxia chambers, an accurate spatial sample translation system is required along with a higher sample irradiation rate. Both of these features are still not optimized for use with laser-driven ions, although these features are already used in practice with cyclotron-accelerated protons. For example on the LNS CATANA beam line, we have previously used a X- and Y-axis sample stage translator to irradiate multiple fields on the same slide flask or several slide flasks in a short time fraction [37, 50, 51]. Some investigators have used an inflatable plastic bag [52, 53] to enclose the samples and keep them hypoxic, however this approach may not be suitable for applications where the samples are irradiated vertically, as in our case with laser driven ions. Also the geometry of the irradiation port and the safety guidelines may not allow the use of such inflatable chamber due to irregular shape and size and the risk that the bag punctures and contents spill out.

For reproducible dosimetry, a thin window membrane was used to minimise any impact of charged particle interactions within the chambers. We used GEANT4 simulation to verify energy and dose changes inside and outside the hypoxia chamber and found no changes as shown in Fig. 2f. Such dosimetry verifications are very important as the aluminium or other components used in chambers upon interaction with protons may lead to significant beam energy attenuation (particularly when working at moderate proton energies) or produce secondary particles, which may contribute to the delivered dose. In the previous studies, the investigators did not comment on whether the material of the chambers attenuated any dose or if there was any significant generation of the secondary particles upon the interaction of charged particles with hypoxia chamber materials [26, 31, 32].

Using the chambers, we tested the effects of hypoxia on key radiobiological endpoints such as DNA DSB damage and cell survival for two cell lines. We used AG01522B primary human fibroblasts as they have been extensively used as radiobiological models to represent normal cells [16, 17, 37, 51–54] and patient-derived glioblastoma E2 stem like cells, as a model system for radioresistant cells which have previously been used to evaluate the role of DNA damage signalling and DNA repair inhibitors in radiation-resistant brain tumours [55–57]. Due to the unavailability of the E2 cells at the time of the laser-driven experiments, we limited the DNA DSB damage assay to AG01522B cells, while for cell survival studies with X-rays we used both cell lines.

The main aim of this paper is to show the potential of the designed chamber to maintain radiobiological hypoxic environment during irradiations. Under normoxic conditions, only background cytoplasmic staining of HIF-1α was observed while upon hypoxia induction for 4 h, an intense nuclear staining of HIF-1α was clearly detected.

53BP1 foci formation is regarded as one of the hallmarks of DNA DSB damage [39–42, 58]. We detected 53BP1 foci formation in AG01522B cells irradiated with 15 MeV protons, with simultaneous staining of HIF-1α, a hypoxia biomarker, by immunofluorescent microscopy as shown in Fig. 5a. The images show the nuclear localisation of the 53BP1 foci in both the hypoxic and oxic samples. The initial DNA DSB damage yield as confirmed through 53BP1 foci at 0.5 h did not vary significantly among the cells irradiated with laser-driven protons, conventional dose rate protons or X-rays under normoxic conditions. This confirms other published data using γ-H2AX foci [15] and both γ-H2AX and 53BP1 [59] for 4.5 MeV [15] or 2.1 MeV [59] laser-driven protons, X-rays or cyclotron-accelerated protons under normoxia. In our study, for the first time we also compare laser-driven proton effects at ultra-high dose-rate under oxic and hypoxic conditions and show that residual DNA DSB damage at 24 h post-irradiation was significantly higher (*P* < 0.05) in hypoxic cells compared to the oxic AG01522B cells. In contrast, the residual DNA DSB damage levels induced by X-rays and conventional dose rate protons, both under the normoxic and hypoxic conditions were similar. So far, at lower dose-rates, (~ 100 Gy/s) studies of FLASH effects have observed these mainly under in vivo conditions [20, 21, 60–63] and only a few investigators have studied them under in vitro conditions [22, 64, 65]. While the normal tissue sparing effects of FLASH irradiation are unique, still no clear mechanisms have been identified for the observed effects. Ultra-high dose rate induced oxygen depletion was hypothesized several decades ago [18] and several investigators have recently observed FLASH effects under reduced oxygen conditions or hypoxia e.g. prostate cancer cells when irradiated at FLASH dose rates under hypoxia [22] showed a higher surviving fractions compared to similar doses at conventional dose rates. Furthermore, upon increasing the oxygen concentration during FLASH irradiation, Montay Gruel et al. observed that the sparing of the neurocognitive functions in mice decreased [9] after irradiation, indicating that a reduced oxygen concentration during irradiation at FLASH dose rates favours tissue sparing.

Recent modelling studies have tested the role of FLASH irradiation-induced oxygen depletion [23, 24, 66–68] and suggest that the radiation doses required to induce radiobiological hypoxia are several magnitudes higher than the dose used for in vivo and in vitro FLASH experiments. However, the model of radiation induced transient oxygen depletion, as suggested by Favaudon et al., seems to be a reasonable explanation for the FLASH effects alongside the differential nature and variations in the bimolecular composition, metabolism, free radicals generation and free radicals clearance mechanisms of the normal and cancer cells [69].

Similar amount of cell killing and DNA DSB damage with electrons at FLASH and conventional dose rates have been observed in cancer and normal cells, as suggested earlier under oxic conditions. In contrast, the higher residual DNA DSB damage under hypoxia observed here with laser-driven protons at ultra-high dose-rates clearly suggests a dose rate effect, which has not so far been reported. As residual DNA DSB damage observed through persistent γ-H2AX foci has been associated with late normal tissue toxicity [70], it is reasonable to infer that the higher residual 53BP1 foci levels measured under hypoxia may result in enhanced cell killing. Enhanced cell killing under hypoxia would clearly lead to improved tumour control as hypoxia is a prominent feature of solid tumours [71] in contrast to normal tissues which may become transiently hypoxic during the irradiation (due to local oxygen depletion if the dose/dose-rate is high enough). This warrants further investigation and confirmation of the yields of DNA DSB damage induced by laser-driven proton under hypoxia, and, for a more comprehensive assessment of the effect, future studies should include comparisons in a wider range of models.

As DNA DSB damage is a key mediator of cell-death, we also tested whether the hypoxic environment inside the chambers has any impact on cell survival. OER is a widely used parameter [27, 72] under both clinical and pre-clinical settings to quantify oxygen sensitisation [73, 74]. We quantified the OER for cell survival of the AG01522B and patient derived E2 GBM stem cells following X-rays irradiation. As shown in Fig. 6a and b the dose response curve of the two cell lines differs due to their intrinsic radiosensitivity. The OER value for the AG01522B cells was 2.2 ± 0.1, lower than that measured for the radioresistant E2 cells ( 2.5 ± 0.1) at D_90_ similar to the OER values reported for various normal and tumour cell lines [75, 76].

Our hypoxia chambers, once gassed, can be used as a standalone unit unlike the previously developed systems, which rely on continuous gassing [31], and can result in a bulky system. Thanks to their portability and bespoke design, the chambers enabled the first radiobiology measurements under controlled oxygen conditions employing laser driven protons, as well as allowing comparator measurements employing conventional dose rate protons and X-rays.

## Conclusions

In this manuscript, we have described the development of a portable hypoxia chamber specifically designed for use in radiobiology experiments in ultra-high dose rates regimes employing beams of laser-driven protons. We have also presented the chamber’s successful application to the study of radiobiological endpoints such as DNA DSB damage and cell survival employing not only laser-driven protons, but also conventional proton and X-ray sources for reference studies. Our results indicate similarities with previously published data but also provide a novel benchmark indicating higher residual DNA DSB damage inflicted by laser-driven UHDR protons under hypoxia compared to cyclotron-accelerated protons.

## Supplementary Information

**Additional file 1**. Supplementary tables and figures.

## Data Availability

Raw data can be available upon request from the Corresponding authors.
