# Supplementary material for: Development of a portable hypoxia chamber for ultra-high dose rate laser-driven proton radiobiology applications
Source: Radiat Oncol. 2022 Apr 15;17:77. doi: 10.1186/s13014-022-02024-3 (PMC9013042; doi:10.1186/s13014-022-02024-3)
Supplement: Supplementary file 1 — Additional file 1. Supplementary tables and figures. [file 13014_2022_2024_MOESM1_ESM.docx]

# Supplementary Figures and Tables

**Table-1 Oxygen Enhancement Ratio for X-rays in AG01522 and E2 cells**

|  | **OER D10** | **Error**  **(SEM)** | **OER D50** | **Error** | **OER D90** | **Error** |
| --- | --- | --- | --- | --- | --- | --- |
| **AG01522** | 1.80 | 0.09 | 2.03 | 0.10 | 2.16 | 0.10 |
|  |  |  |  |  |  |  |
| **E2 Cells** | 1.84 | 0.09 | 2.24 | 0.11 | 2.50 | 0.12 |
|  |  |  |  |  |  |  |

Table-1. OER values obtained at various doses resulting in 10%, 50 % and 90% cell survival after exposure to X-rays under oxic or hypoxic conditions.

**Table-2. Oxygen Concentration vs Time for various Oxygen concentrations**

| **% Oxygen** | **Time in minutes after disconnecting from gassing** |
| --- | --- |
| 0.2 | 36 |
| 0.4 | 60 |
| 1 | 240 |
| 2 | 480 |
| ~4 | 1440 |

Table-2 showing the concentration of oxygen (%) inside chambers after gassing for 15 minutes and then disconnecting from the gas supply (95% Nitrogen and 5% CO_2_) and the time upto which concentration is maintained within chamber.

# Additional Figure-1


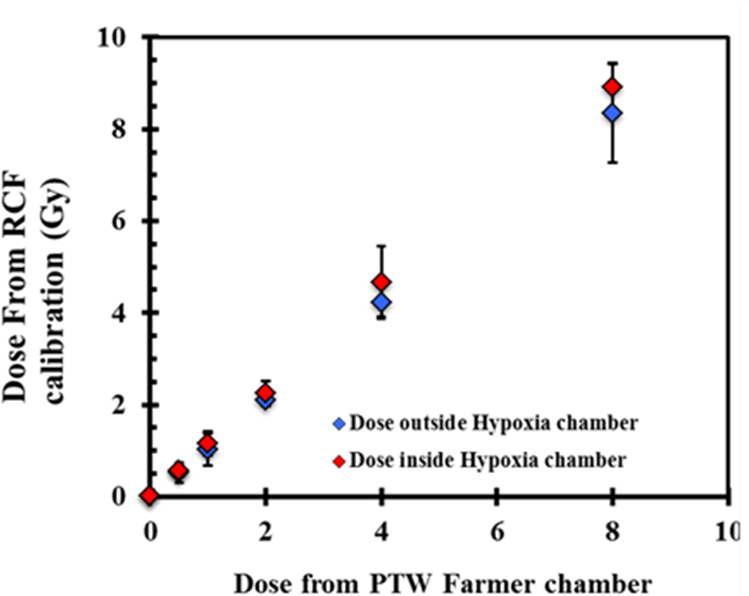


Additional Figure 1. X-rays dose measurements inside and outside the hypoxia chambers.

The dose was calculated from the EBT films paced inside the hypoxia chambers at the cell position and exposed to 225 kVp X-rays. Dose outside the chamber was measured on EBT 3 film placed on the PVDC window.The error bars represent the SD of three independent replicates.

# Additional Figure 2

**Relative foci induction in AG01522 cells**


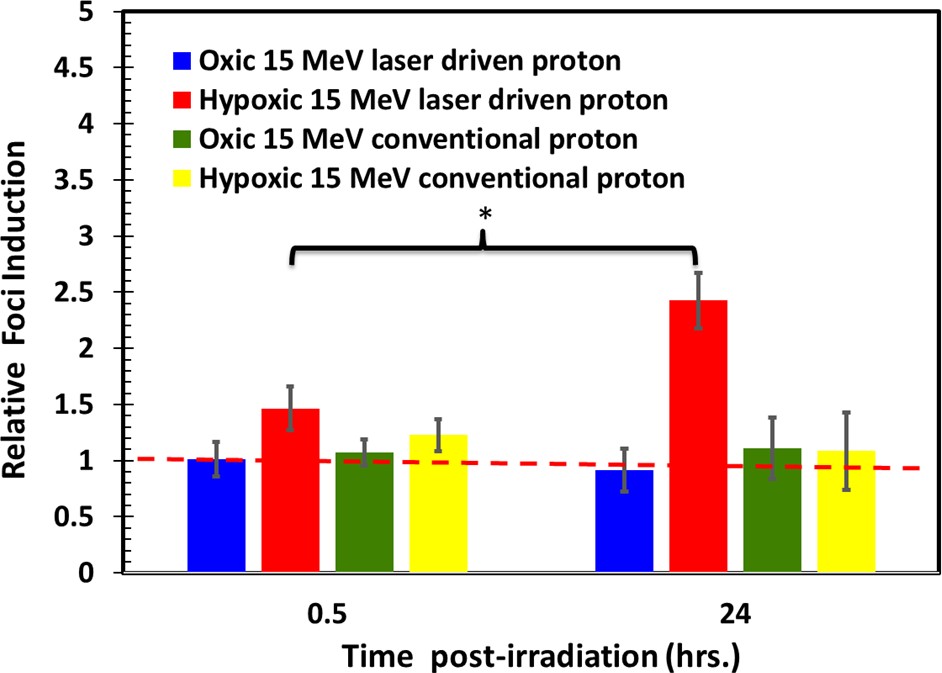


**Additional Figure 2**. Relative 53BP1 foci induction values 53BP1 foci induced by laser- driven protons and conventional protons under oxic and hypoxic conditions. The residual foci induced by laser-driven protons under hypoxic conditions clearly show an increased relative foci induction value
